# Intracerebral Hemorrhage with Intraventricular Extension—Getting the Prognosis Right Early

**DOI:** 10.3389/fneur.2017.00418

**Published:** 2017-08-17

**Authors:** Christoph Stretz, Catherine Gao, David M. Greer, Caitlin Loomis, Emily J. Gilmore, Adam J. Kundishora, Charles C. Matouk, David Y. Hwang

**Affiliations:** ^1^Division of Neurocritical Care and Emergency Neurology, Department of Neurology, Yale School of Medicine, New Haven, CT, United States; ^2^Department of Medicine, Yale School of Medicine, New Haven, CT, United States; ^3^Department of Neurology, Boston University School of Medicine, Boston, MA, United States; ^4^Division of Vascular Neurology, Department of Neurology, Yale School of Medicine, New Haven, CT, United States; ^5^Department of Neurosurgery, Yale School of Medicine, New Haven, CT, United States; ^6^Department of Radiology and Biomedical Engineering, Yale School of Medicine, CT, United States

**Keywords:** intracerebral hemorrhage, intraventricular hemorrhage, prognosis, outcome, self-fulfilling prophesy

## Abstract

**Background:**

Early accurate outcome prognostication for patients with intracerebral hemorrhage (ICH) and accompanying intraventricular hemorrhage (IVH) is often challenging (1). Acute hydrocephalus often contributes to a poor clinical exam (2) and can portend significant morbidity and mortality (3). Accordingly, the inpatient neurologist may feel inclined to recommend limitations of care for an ICH patient admitted with a large IVH burden and poor exam.

**Case presentation:**

We present a patient with significant IVH and minimal ICH who deteriorated rapidly to coma after presentation. Despite this exam, an initially non-functioning diversion of cerebrospinal fluid (CSF) and temporary halt of further attempts of CSF diversion in the setting of an early “do not resuscitate order,” our patient gradually improved and, with supportive ICU care and rehabilitation, was able to communicate and ambulate with assistance at 12 weeks.

**Conclusion:**

Patients with ICH with IVH do have the capacity to improve dramatically even with relatively conservative management. Unless previous limitations of care exist, we recommend that early judgments of prognosis for patients with ICH and/or IVH should be delayed for at least 72 h until the patient’s clinical trajectory over time is better understood.

## Background

Early, accurate outcome prognostication for patients with intracerebral hemorrhage (ICH) and accompanying intraventricular hemorrhage (IVH) is often challenging (1). IVH is present initially in one quarter of patients with ICH or may occur as subsequent extension of the ICH component (2). Acute hydrocephalus often contributes to a worsened clinical exam (2) and can portend significant morbidity and mortality (3). Based on these teachings, the inpatient neurologist may feel inclined to recommend limitations of care for an ICH patient admitted with a large IVH burden and poor exam. Here, we report on a patient with IVH and minimal ICH who made a remarkable recovery despite an initially devastating exam and temporary pause in additional cerebrospinal fluid (CSF) diversion in the setting of a non-functioning external ventricular drainage (EVD) and transient code status of “Do not resuscitate.”

## Case Presentation

A 56-year-old woman with a history of atrial fibrillation, hypertension, congestive heart failure, and mechanical mitral valve repair following rheumatic heart disease on warfarin was transferred to our hospital after being found unresponsive. On arrival, her blood pressure was 141/66. She was awake and able to move all four extremities to command; however, she had no verbal output, and her Glasgow Coma Scale (GCS) was 10 (E = eye opening 3, V = verbal response 1, and M = motor response 6).

**Figure 1:**
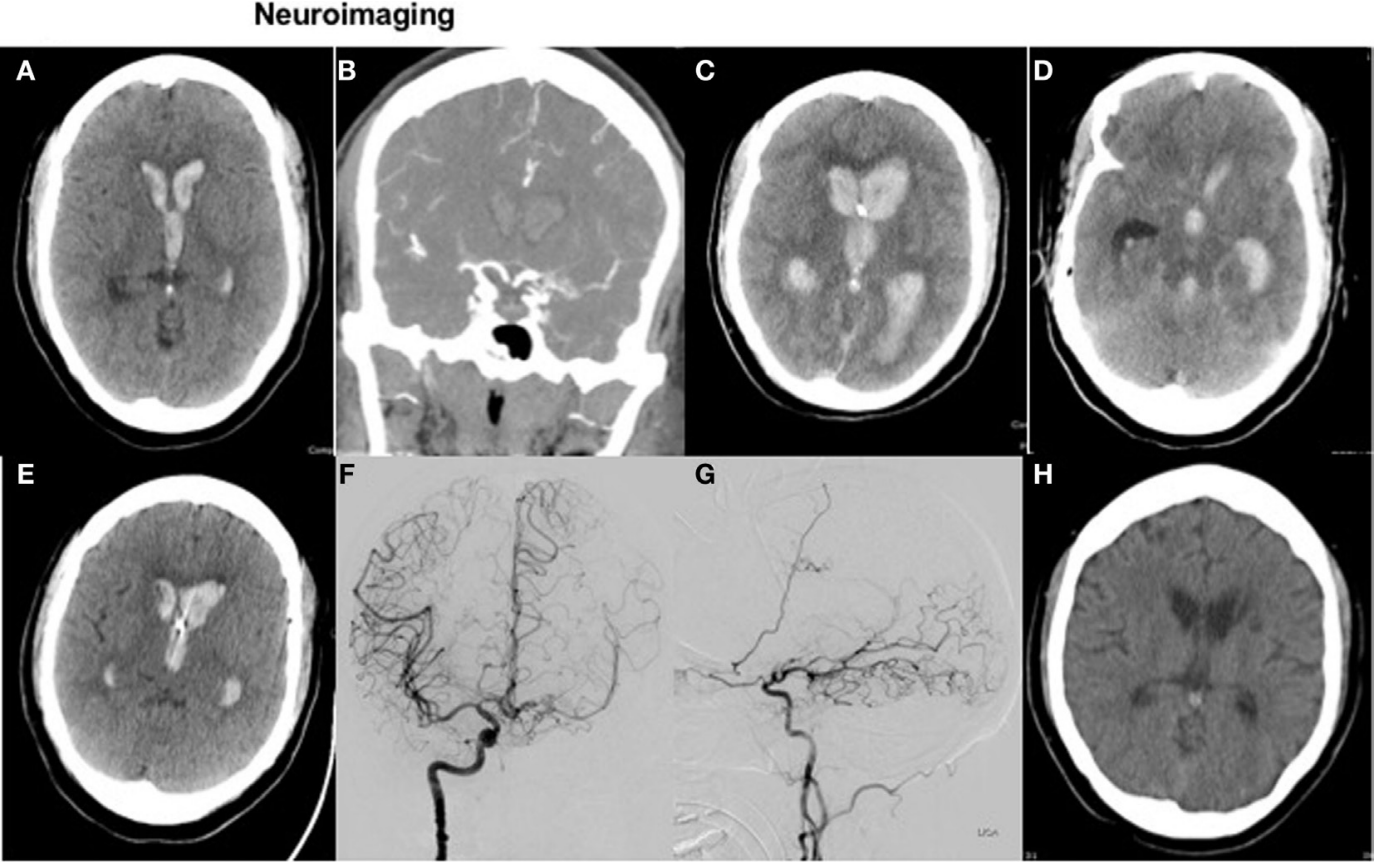
Neuroimaging. Figure 1 with axial CT head **(A)** and coronal CTA **(B)** on presentation, with repeat axial CT 3 h later **(C,D)** and axial images on posthemorrhage day 4 and 21 **(E,H)**. Diagnostic cerebral angiogram (coronal view and sagittal view) is shown in images **(F,G)**, respectively.

A head CT (Figure 1A) demonstrated a small ICH in the left periventricular white matter with significant intraventricular blood. CT angiogram of the head (Figure 1B) was suggestive of Moyamoya-like vasculopathy involving the left internal carotid artery (ICA) summit. Laboratory testing showed an INR of 3.6. She received intravenous vitamin K and prothrombin complex concentrate for INR reversal.

Following intubation for airway protection due to declining mental status, an EVD was placed; however, drainage ceased within 1 h of placement due to obstruction of the catheter with blood. On repeat clinical exam off sedation, the patient was not following commands with a GCS of 4T. She had fixed, non-reactive pupils at 3 mm; absent corneal, oculocephalic, cough, and gag reflexes; no spontaneous breaths; and slight flexion to painful stimuli in the upper extremities. Repeat head CT showed worsened IVH (Figure 1C) with hydrocephalus and apparent compression of the midbrain (Figure 1D).

The patient’s husband was immediately informed that these developments were concerning for a devastating functional outcome, if she were to survive. Consequently, no further attempt was made to adjust the EVD for improved drainage or place a second EVD. Her code status was changed to “do not resuscitate” in the evening of admission. Her husband requested time to consider full comfort measures as goals of care, while continued routine ICU care would be pursued. Given the non-functioning EVD, obtained ICP readings were deemed inaccurate and thus not further acted upon through EVD manipulation or osmotherapy.

However, the patient’s clinical exam spontaneously improved over the following 48 h, while supportive ICU care was continued. She began overbreathing the ventilator and regained reactive pupils, cough, and gag reflexes, with absent, but eventual return of corneal and oculocephalic reflexes. On motor exam, she extended in the right arm and leg, but moved the left arm spontaneously and withdrew in both left arm and leg.

Shortly after these observations, her EVD then started to drain spontaneously, without intervention, again approximately 48 h after initial placement. Based on this clinical improvement, the patient’s husband requested that her code status be changed back to “full resuscitation.” Repeat head CT on postbleed day 4 (Figure 1E) showed improved hydrocephalus, although still with ventricles “casted” from IVH. A more aggressive care approach was pursued at that time, and a left-sided EVD was placed.

A diagnostic cerebral angiogram confirmed a Moyamoya syndrome characterized by (1) chronic occlusion of the left supraclinoid ICA above the origin of the anterior choroidal artery, (2) a meshwork of small vessels replacing the ICA summit, proximal ACA, and proximal MCA, and (3) robust collaterals supplying the L MCA vascular territory *via* a widely patent anterior communicating artery and leptomeningeal collaterals derived from the distal L PCA (Figures 1F,G). No definite, angiographically demonstrable culprit lesion was demonstrated. However, in light of these findings, empiric administration of intraventricular tissue plasminogen activator was deferred, given an unknown further risk of intracranial hemorrhage in the setting of the patient’s unique cerebrovascular anatomy. The patient was extubated safely 1 week after admission.

At the time of discharge to inpatient rehabilitation, 3 weeks posthemorrhage, the patient was able to follow commands intermittently and could move the right arm in the plane, the right leg with flicker movement, and the left arm and leg antigravity.

Figure 1H shows a surveillance head CT at 3 weeks without residual intraventricular blood.

At the time of outpatient follow-up at 12 weeks, the patient was alert, communicated well, was able to eat by herself, and required two-person assist for walking. Neurologic examination showed her to be partly oriented, with full ability to follow commands and no evidence of aphasia. On cranial nerve testing, she could count fingers in central visual fields only. Gaze was dysconjugate with a right third nerve palsy and bilateral restricted upgaze. The remaining cranial nerves were intact. Motor exam showed near full strength in the upper extremities and strength of 4 of 5 in the lower extremities. Sensory and cerebellar functions were intact. Her mobility had further improved, and at 9 months, she is able to ambulate with a rolling walker and requires one-person assist at times.

## Discussion

Nearly 30% of patients who die of ICH in a hospital have aggressive care withdrawn within the first 24 h of admission (4). The possibility that self-fulfilling prophecies may influence the care of ICH patients heavily has been much debated in literature (5). IVH and hydrocephalus in particular can contribute to clinical exam deterioration for an ICH patient (2) and may in some cases lead clinicians to assume early on during a hospital admission that prognosis will inevitably be poor.

These assumptions may be rooted in historical IVH outcome data. While a detailed discussion of the differences between primary and secondary IVH is beyond this case report, it deserves mention that several studies have shown a worse outcome with secondary IVH (6).

Intraventricular hemorrhage and hydrocephalus in the setting of ICH have been traditionally associated with worse ICH outcomes in multiple cohort studies. A cohort study of 129 medically managed patients with supratentorial ICH published in 1999 found a mortality rate of 43% for patients with ICH and IVH versus 9% for those patients with ICH alone (7). The ICH score, the most widely used and validated clinical grading scale for ICH since its publication in 2001, specifically incorporates the presence of intraventricular blood as a factor contributing to poorer prognosis (8). The modified Graeb Scale, a semiquantitative method for assessing IVH extension, for ICH patients has also been shown to be an independent predictor of 30-day mortality and poor functional outcome (9).

There is ambiguity regarding the association of timing of IVH with a poor outcome: Witsch et al. (10) found that early, but not delayed IVH was associated with a poor outcome (10). Delayed IVH was defined as IVH that was present on the subsequent, but not initial head CT (10). On the contrary, Maas et al. (11) found delayed IVH to be an independent predictor of short-term mortality and poor functional outcome at 3 months (11).

However, it should be noted that hydrocephalus was not corrected for as a potential contributor to poor outcome. In a retrospective observational study, Mahta et al. (12) found that IVH in a patient with ICH in the absence of hydrocephalus was not associated with increased mortality or disability (12).

The assumption among clinicians of poor outcome in patients with massive intraventricular extension of ICH may in and of itself influence early limitation of care patterns. In the 1999 study mentioned above that cited a 43% mortality rate for ICH patients with IVH, only 2 of the 47 patients with IVH had EVDs placed, and no intraventricular thrombolytics were administered (7). In a separate, more recent cohort of greater than 270 ICH inpatients, an EVD was also found to be rarely placed, despite the fact that almost 50% of patients had hydrocephalus on the initial CT scan (13). In search for more accurate prognostic factors determining outcome in patients with ICH, Maas et al. (14) found that the GCS score on day 5 (“GCS 5”) outperformed the ICH score (8) in predicting a good (mRS 0–3) versus poor (mRS 4–6) outcome at 3 months.

Most recently, the CLEAR III trial (15), while unable to prove a definitive difference in functional outcomes between IVH patients treated with intraventricular alteplase versus placebo, did reveal more favorable outcomes overall than expected; in fact, 45% of the patients in the control group for this trial achieved the ability to walk, whereas the initial power calculations based on historical data estimated that no more than 22% in the control group would achieve this outcome (16). Furthermore, for all ICH patients (with or without IVH), avoidance of early limitations of care in the hospital is recommended by the American Heart Association (AHA) Guidelines on the Management of Spontaneous ICH (17) and has been shown as a treatment strategy to lower mortality rates compared to those rates that might be predicted by the ICH score (8) and other prognostic models with limitations of care bias incorporated.

In addition to the AHA statement, the Neurocritical Care Society also recommends treating patients with devastating brain injury, such as ICH with IVH, for at least 72 h before deciding on limitation of life-sustaining therapy (18). For IVH patients in particular, we share the opinion expressed in the AHA and NCS guidelines that, unless clear advance directives exist, early withdrawal of life-sustaining therapy should be deferred as a default plan of management, in particular when intracerebral and/or intraventricular hematomas are amenable to surgical evacuation and/or CSF diversion (19). Further, potential confounders of a poor exam such as infection, sedative adverse effects of medications, and non-convulsive seizures should be taken into account and addressed.

The case presented in this report is an example of the outcome that an ICH patient with a large amount of IVH can achieve even when the medical team initially decides to place limitations on therapy. In hindsight, the first discussion with the patient’s husband on goals of care occurred very early in the setting of the patient’s deterioration to a comatose state and imaging findings of hydrocephalus and apparent brainstem injury. It is unclear if foregoing this initial discussion and addressing the non-functioning EVD could have contributed to an improved neurologic outcome.

This case complements emerging data that the natural history for certain ICH patients with large IVH may not be as poor as one may expect. While prognostic factors for a poor outcome such as the GCS on day 5 in the study by Maas et al. (14) are certainly desirable to aid in decision-making, they should be validated in prospective multicentric studies. Observational data derived from a cohort, such as in the study presented, should always be used with caution as a decision-making tool for the individual patient, taking into account specific comorbidities and potential confounding factors.

Our case exemplifies that a poor neurologic exam can be mediated by minimal primary ICH but with large IVH. While our patient’s IVH would be classified as secondary, the primary ICH component likely is a minor contributor to her outcome. This represents a stark contrast to the patient population in the study by Tuhrim et al. (7), where intraventricular extension of ICH was associated with more than twice as large baseline hematoma volume and consequently a higher mortality rate (43% with IVH extension versus 9% without associated IVH).

Thus, potential explanations for our patient’s remarkable recovery are minimal parenchymal injury, aggressive CSF diversion, and supportive ICU care—although temporarily halted—which were provided despite a poor neurologic exam upon admission to the ICU.

## Conclusion

Patients with ICH and IVH do have the capacity to improve dramatically even with relatively conservative management. This case serves as an example of significant and spontaneous clinical improvement for an ICH patient with massive IVH and hydrocephalus, despite a poor initial neurologic examination, devastating initial imaging, an initially non-functioning EVD, and an early attempt by a medical team to withdraw care early on during the hospitalization. We recommend that, in the absence of established limitations of care prior to presentation, early judgments of prognosis for patients with ICH and IVH be delayed for at least 72 h until the individual patient’s clinical trajectory over time is better understood.

## Ethics Statement

No investigation or intervention was performed outside routine clinical care for this patient. As this is a case report, without experimental intervention into routine care, no formal research ethics approval is required. Written, fully informed consent was given by the patients’ husband by telephone and recorded for the patient’s anonymized health information to be published in this case report.

## Author Contributions

CS, CG, CL, EG, AK, CM, and DH were involved with the workup and clinical care of the patient. CS, CG, DG, CL, EG, AK, CM, and DH took part in the acquisition and interpretation of data, the draft and critical revision for intellectual content, gave final approval of the version to be published, and agree to be accountable for all aspects of the work in ensuring that questions related to the accuracy or integrity of any part of the work are appropriately investigated and resolved.

## Conflict of Interest Statement

CS, CG, AK, CL, and CM report no disclosures. DG receives a stipend as editor-in-chief for Seminars in Neurology and personal compensation for medical-legal work, not pertinent to the manuscript presented. EG has received funding from Yale’s Center for Clinical Investigation, CTSA Grant (ULTR000142), Yale’s Claude D. Pepper Older Americans Independence Center (P30AG021342 NIH/NIA), the American Brain Foundation, and the National Institutes of Health. DH has received funding from the American Brain Foundation, the Apple Pickers Foundation, the Neurocritical Care Society, and the National Institute on Aging (for Loan Repayment). He has also received recent speaking fees from Pennsylvania State University, Mayo Clinic, and the Society of Critical Care Medicine and modest book royalties from Oxford University Press and the Neurocritical Care Society. None of these funding sources or speaking/writing fees are tied to work presented in this manuscript.

## References

[1] Chu SY Hwang DY. Predicting outcome for intracerebral hemorrhage patients: current tools and their limitations. Semin Neurol (2016) 36(3):254–60. 10.1055/s-0036-1581992 27214700

[2] Morawo AO Gilmore EJ. Critical care management of intracerebral hemorrhage. Semin Neurol (2016) 36(3):225–32. 10.1055/s-0036-1581991 27214697

[3] Dey M Jaffe J Stadnik A Awad IA. External ventricular drainage for intraventricular hemorrhage. Curr Neurol Neurosci Rep (2012) 12:24–33. 10.1007/s11910-011-0231-x 22002766 PMC6777952

[4] Zurasky JA Aiyagari V Zazulia AR Shackelford A Diringer MN. Early mortality following spontaneous intracerebral hemorrhage. Neurology (2005) 64:725–7. 10.1212/01.WNL.0000152045.56837.58 15728302

[5] Becker KJ Baxter AB Cohen WA Bybee HM Tirschwell DL Newell DW Withdrawal of support in intracerebral hemorrhage may lead to self-fulfilling prophecies. Neurology (2001) 56(6):766–72. 10.1212/WNL.56.6.766 11274312

[6] Cucchiara BL Intraventricular hemorrhage. UpToDate (2017). Available from: http://www.uptodate.com/contents/intraventricular-hemorrhage (accessed July 2017).

[7] Tuhrim S Horowitz DR Sacher M Godbold JH Volume of ventricular blood is an important determinant of outcome in supratentorial intracerebral hemorrhage. Crit Care Med (1999) 27:617–21. 10.1097/00003246-199903000-00045 10199544

[8] Hemphill JC Bonovich DC Besmertis L Manley GT Johnston SC. The ICH score: a simple, reliable grading scale for intracerebral hemorrhage. Stroke (2001) 32(4):891–7. 10.1161/01.STR.32.4.891 11283388

[9] Hansen BM Morgan TC Betz J Sundgren PC Norrving B Hanley DF Intraventricular extension of supratentorial intracerebral hemorrhage: the modified Graeb scale improves outcome prediction in Lund stroke register. Neuroepidemiology (2015) 46:43–50. 10.1159/000442575 26668048 PMC6139030

[10] Witsch J Bruce E Meyers E Velazquez A Schmidt JM Suwatcharangkoon S Intraventricular hemorrhage expansion in patients with spontaneous intracerebral hemorrhage. Neurology (2015) 84:989–94. 10.1212/WNL.0000000000001344 25663233 PMC4352099

[11] Maas MB Nemeth AJ Rosenberg NF Kosteva AR Prabhakaran S Naidech AM Delayed intraventricular hemorrhage is common and worsens outcome in intracerebral hemorrhage. Neurology (2013) 80(14):1295–9. 10.1212/WNL.0b013e31828ab2a7 23516315 PMC3656461

[12] Mahta A Katz PM Kamel H Azizi SA. Intracerebral hemorrhage with intraventricular extension and no hydrocephalus may not increase mortality or severe disability. J Clin Neurosci (2016) 30:56–9. 10.1016/j.jocn.2015.11.028 26972705

[13] Zahuranec DB Brown DL Lisabeth LD Gonzales NR Longwell PJ Smith MA Early care limitations independently predict mortality after intracerebral hemorrhage. Neurology (2007) 68(20):1651–7. 10.1212/01.wnl.0000261906.93238.72 17502545

[14] Maas MB Francis BA Sangha RS Lizza BD Liotta EM Naidech AM. Refining prognosis for intracerebral hemorrhage by early reassessment. Cerebrovasc Dis (2017) 43:110–6. 10.1159/000452679 28049196 PMC5380575

[15] Hanley DF Lane K McBee N Ziai W Tuhrim S Lees KR Thrombolytic removal of intraventricular haemorrhage in treatment of severe stroke: results of the randomised, multicentre, multiregion, placebo-controlled CLEAR III trial. Lancet (2017) 389:603–11. 10.1016/S0140-6736(16)32410-2 28081952 PMC6108339

[16] Rabinstein A Intracerebral hemorrhage – no good treatment, but treatment helps. Lancet (2017) 389(10069):575–6. 10.1016/S0140-6736(17)30002-8 28081951

[17] Hemphill JC III Greenberg SM Anderson CS Becker K Bendok BR Cushman M Guidelines for the management of spontaneous intracerebral hemorrhage: a guideline for healthcare professionals from the American Heart Association/American Stroke Association. Stroke (2015) 46(7):2032–60. 10.1161/STR.0000000000000069 26022637

[18] Souter MJ Blissitt PA Blosser S Bonomo J Greer D Jichici D Recommendations for the critical care management of devastating brain injury: prognostication, psychosocial, and ethical management: a position statement for healthcare professionals from the neurocritical care society. Neurocrit Care (2015) 23(1):4–13. 10.1007/s12028-015-0137-6 25894452

[19] Morgenstern LB Zahuranec DB Sánchez BN Becker KJ Geraghty M Hughes R Full medical support for intracerebral hemorrhage. Neurology (2015) 84(17):1739–44. 10.1212/WNL.0000000000001525 25817842 PMC4424123

